# Efficacy and safety of adjunctive antiseizure medications for dravet syndrome: A systematic review and network meta-analysis

**DOI:** 10.3389/fphar.2022.980937

**Published:** 2022-08-31

**Authors:** Jianhua Wu, Liu Zhang, Xi Zhou, Jiajun Wang, Xiangyi Zheng, Hankun Hu, Dongfang Wu

**Affiliations:** Department of Pharmacy, Zhongnan Hospital of Wuhan University, Wuhan, China

**Keywords:** soticlestat, stiripentol, fenfluramine, cannabidiol, dravet syndrome

## Abstract

**Purpose:** Recently, the U.S. Food and Drug Administration (FDA) approved stiripentol, cannabidiol, and fenfluramine to treat patients with Dravet syndrome (DS). Moreover, soticlestat was determined as a promising new drug for the treatment of DS as it has good efficacy and safety. However, the efficacy and safety of these drugs have not yet been evaluated in “head-to-head” trials. This study aimed to compare and evaluate the efficacy and safety of these adjunctive antiseizure medications in the treatment of DS.

**Methods:** We searched in PubMed, Embase, Cochrane Library, and Web of Science databases for randomized controlled trials (RCTs) and open-label extension (OLE) studies in patients with DS. We performed a random-effect meta-analysis of OLE studies and a network meta-analysis for RCTs to evaluate the efficacy and safety of antiseizure medications in the treatment of DS. Primary efficacy outcomes were defined as a ≥50% reduction in seizure frequency compared with baseline. Furthermore, safety evaluation indicators were defined as the incidence of adverse events (AEs) and serious adverse events (SAEs) during treatment. Relative ranking was assessed using the surface under the cumulative ranking curve (SUCRA) probabilities.

**Results:** Seven RCTs involving four antiseizure medications (stiripentol, cannabidiol, fenfluramine, and soticlestat) and a total of 634 patients were included in the analysis. According to the SUCRA results, all four drugs significantly reduced the frequency of seizures compared with the placebo. Soticlestat was the most likely to reduce seizure frequency by ≥50% compared to the baseline [risk ratio (RR): 19.32; 95% confidence interval (CI): 1.20–311.40], followed by stiripentol and fenfluramine. Stiripentol was ranked highest for the near percentage reduction in the seizure rate from baseline [RR: 12.33; 95% CI: 1.71–89.17] and the occurrence of any treatment-emergent adverse events [RR: 3.73; 95% CI: 1.65–8.43] and serious adverse events [RR: 4.76; 95% CI: 0.61–37.28]. A total of ten OLE studies containing 1,121 patients were included in our study. According to the results of the meta-analysis, the order of probability of reducing seizure frequency by ≥50% was fenfluramine (0.715, 95% CI: 0.621–0.808), stiripentol (0.604, 95% CI: 0.502–0.706), cannabidiol (0.448, 95% CI: 0.403–0.493). And the probability of occurrence of AEs is ranked as fenfluramine(0.832, 95% CI: 0.795–0.869), cannabidiol (0.825, 95% CI:0.701–0.950), stiripentol (0.823, 95% CI: 0.707–0.938), soticlestat (0.688, 95% CI: 0.413–0.890).

**Conclusion:** According to the results of indirect comparison of efficacy and safety, cannabidiol is slightly inferior to the other three antiseizure medications in terms of efficacy and safety. Soticlestat, fenfluramine, and stripentol may have little difference in efficacy, but soticlestat and fenfluramine are safer. Soticlestat is probably the best adjunctive antiseizure medication, followed by fenfluramine. This conclusion is consistent with the comparison of long-term efficacy and safety.

## Introduction

Dravet syndrome (DS) is a treatment-resistant, developmental, and genetic form of epileptic encephalopathy (Mei et al., 2019; Lo et al., 2021). The main features of DS include an early onset, various forms of seizures, delayed psychomotor development, and drug resistance (Dravet, 2011). Dravet syndrome progression is divided into three stages: heating, deterioration, and stability (Wheless et al., 2020). The overall incidence of DS is approximately 1/40,900–1/22,000, accounting for approximately 29.5% of various types of myoclonic epilepsy in children (Brunklaus et al., 2012; Bayat et al., 2015; Wu et al., 2015; Bjurulf et al., 2022). Studies have found that DS is related to mutations in the *SCN1A* gene, which encodes the NaV1.1 channel, and more than 85% of patients have mutations in this gene (Claes et al., 2001). These changes make the channels of inhibitory interneurons less functional, which can overexcite neurons, leading to seizures (Catterall et al., 2010). There are many treatment options for DS (Ziobro et al., 2018). However, antiseizure medication treatment remains the mainstay treatment for DS (Chin et al., 2021). Valproic acid and clobazam are the first-line drugs for treating DS according to the practical guidelines for treating DS with anti-seizure medication (Strzelczyk and Schubert-Bast, 2022). Medications that may exacerbate seizures, including sodium channel inhibitors (e.g., carbamazepine, oxcarbazepine, lamotrigine, and phenytoin) and the γ-aminobutyric acid (GABA) transaminase inhibitor vigabatrin, should be avoided (Wirrell et al., 2017). However, most patients with DS exhibit drug resistance, which leads to deficient treatment effects on epilepsy (Devi et al., 2021). In such cases, multiple drugs must be used simultaneously. Recently, the U.S. Food and Drug Administration (FDA) approved medications for DS, which included stiripentol, fenfluramine, and cannabidiol (Sharawat et al., 2021). Moreover, it was found that soticlestat, a new drug used to treat DS, showed promising efficacy and safety in a recently completed phase 2 clinical trial (Strzelczyk and Schubert-Bast, 2022). These drugs act through different mechanisms. Stiripentol affects the metabolism of other antiseizure medications such as clobazam by enhancing central γ-aminobutyric acid transmission and inhibiting several P450 cytochromes (Devi et al., 2021). Cannabidiol reduces neuronal excitability through inhibition of adenosine transport and modulation of intracellular Ca2^+^(Gray and Whalley, 2020). Fenfluramine enhances the effects of 5-hydroxytryptamine to exerts antiepileptic activity (Sourbron et al., 2017). Fenfluramine is also a positive regulator of the sigma-1 receptor (Martin et al., 2020). Soticlestat is a cholesterol 24-hydroxylase inhibitor that can reduce the level of 24S-hydroxycholesterol (24HC) (Hawkins et al., 2021; Koike et al., 2021). There is no direct head-to-head study on the efficacy and safety of these four drugs, and clinical decision-making largely depends on the availability of drugs in different regions, patient characteristics and needs, and personal preferences, making it difficult for clinicians to choose the best treatment method (Zhang et al., 2022). Therefore, this study aimed to compare and evaluate the efficacy and safety of currently available DS treatment medications by performing a network meta-analysis of randomized controlled trials (RCTs), and to evaluate the long-term efficacy and safety of antiseizure medications by performing a meta-analysis of open-label extension (OLE) studies, to provide a reference for the selection of clinical treatment medications for DS.

## Materials and mehthods

The conduct and reporting of this study followed the Preferred Reporting Items for Systematic Reviews (Page et al., 2021) and the PRISMA Extension Statement for Reporting of Systematic Reviews Incorporating Network Meta-analyses (Hutton et al., 2015), using standardized protocols for review and data extraction. Furthermore, they were registered in PROSPERO. The primary outcome measure for efficacy was the number of patients with a ≥50% reduction in seizure frequency from the baseline and a nearly 100% reduction in seizure frequency from the baseline. Seizures included tonic, epileptic spasms, tonic-spasmodic, and atonic seizures (Golub and Reddy, 2021). The primary safety outcome measures were the incidence of adverse events (AEs) and serious adverse events (SAEs). Adverse reactions mainly include somnolence, decreased appetite, and diarrhea (Lagae et al., 2019; Lattanzi et al., 2021). The effect size was defined as the risk ratio (RR) and prevalence of events. A meta-analysis and a network meta-analysis of efficacy and safety indicators were performed according to the frequentist theory and a consensus model.

### Search strategy and selection criteria

The following electronic databases were searched from the date of their inception to 3 March 2022: PubMed, Embase, the Cochrane Library, and Web of Science, without any restrictions on age, setting, sex, ethnicity, or publication year. The search keywords used included “Dravet syndrome,” “severe myoclonic epilepsy of infancy,” “SMEI,” “Soticlestat,” “Stiripentol,” “Fenfluramine,” “Cannabidiol,” “antiseizure medication,” “treatment,” and “seizure”. The searches were combined using Boolean operators (OR, AND), medical subject headings, free text phrases, and variations. We looked through ClinicalTrials.gov, 2018 to find ongoing and unpublished research. To identify other potentially overlooked studies, an additional manual search of the references in the selected trials was included, and systematic reviews were performed.

The inclusion criteria used to find literature were as follows: RCTs, OLE studies, or post-commercial studies; participants diagnosed with DS based on a clinician’s opinion; detailed results were reported for ≥50% and nearly 100% reductions in seizure rates from baseline, AEs, and SAEs; and the efficacy and safety of any dose of these four antiseizure medications were studied and compared with other interventions or placebo. We excluded case reports, case series, retrospective studies, cohort studies, case-control studies, studies published in languages other than Chinese or English, reviews of case reports, review articles, and studies investigating mixed groups of patients, such as DS and Lennox–Gastaut syndrome, unless the articles provided data for the DS group.

### Data extraction and quality assessment

Two investigators independently extracted data from the included literature, including the following fields: author’s name, year of publication, intervention (including antiseizure medications and doses), number of participants in the intervention and control groups, the age and sex of participants, basic information, study time, safety, and efficacy outcome indicators. If the study involved the use of the same drug in different dose groups, these were combined into the same group for the data analysis. The quality of the included studies was assessed using the Cochrane Collaboration Tools to assess the risk of bias using Review Manager 5.4 (Cochrane Collaboration) software. Each study was classified as having a high risk of bias, low risk of bias, or unclear risk of bias according to the following criteria: random sequence generation, allocation concealment, blinding of outcome participants and personnel, blinding of outcome assessment, incomplete outcome data, selection of sexual reporting, and other biases. Two investigators independently assessed the quality of the eligible studies, and disagreements were discussed and resolved by a third investigator.

### Data integration and analysis

This study applied frequentist theory to conduct a network meta-analysis to compare the effectiveness and safety of each intervention across all studies. Since the RCTs were a short-term experiment and the results have certain limitations, we also included relevant OLE studies and post-commercial studies for meta-analysis to compare the long-term efficacy and safety of antiseizure medications. A network evidence graph was used to visualize comparisons of the different interventions, where the size of the nodes represented the number of study participants and the width of the connecting lines represented the number of studies for each drug. Effect estimates for predefined outcomes were expressed as RR with 95% confidence intervals (CIs). Because the included studies were designed to directly compare the treatment with the placebo and there was a lack of direct comparisons between drugs, an inconsistency test was not performed. Multiple doses (e.g., 10 and 20 mg) were included in the treatment, which were combined into one result. Treatments were ranked according to their efficacy and safety according to the area under the surface of the cumulative ranking curve (SUCRA). All statistical analyses were performed using Stata 16.0 (StataCorp, LP College Station, TX, United States).

## Result

### Literature search

**FIGURE 1:**
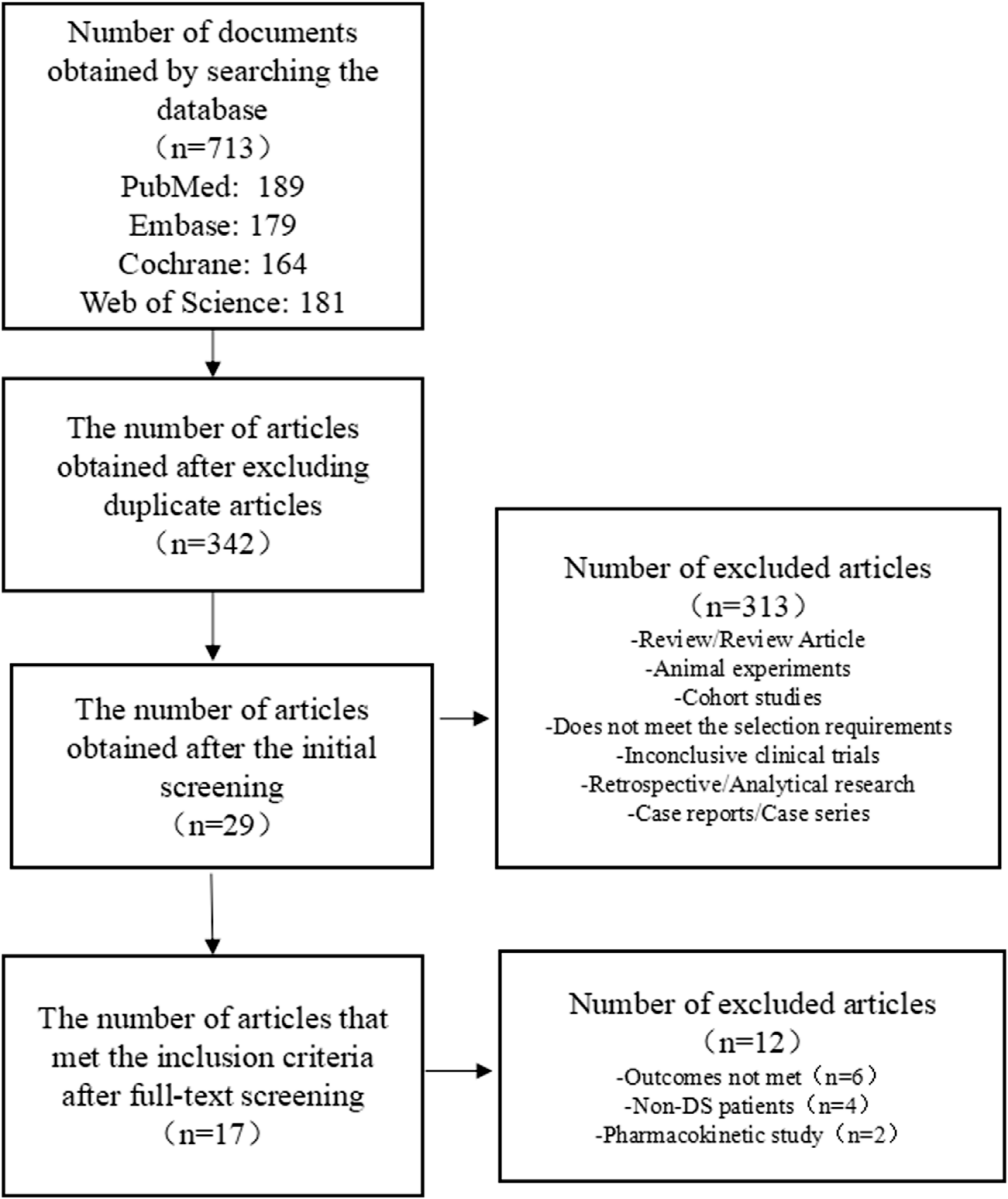
Flow chart of literature selection.

We preliminarily identified 713 studies, of which we excluded 371 repeated studies. A further 313 studies were excluded as these were review/review articles, animal experiments, cohort studies, clinical trials that did not meet the requirements for topic selection, clinical trials with no results, case reports, case series, and retrospective/analytical studies. After a full-text review, 12 studies that did not meet the requirements were excluded from the remaining 29 studies. Seven RCTs comprising 634 patients and ten OLE studies comprising 1,121 patients met the inclusion criteria for this research. A flowchart of the literature selection process is shown in Figure 1.

### Characteristics and quality of the included studies

The main characteristics of the included OLE studies are presented in Table 1. In our study, three studies on stiripentol were included (Inoue et al., 2009; Inoue and Ohtsuka, 2015; Myers et al., 2018), three studies on fenfluramine were included (Specchio et al., 2020; Sullivan et al., 2020; Bishop et al., 2021), three studies on cannabidiol were included (Devinsky et al., 2019; Iannone et al., 2021; Scheffer et al., 2021), and only one study on soticlestat (Halford et al., 2021), a total of 1,121 patients were included. Since the included OLE studies were all single-arm experiments, a network meta-analysis was not possible. Although the soticlestat study only reported safety outcomes and the sample size was small, because it is a promising new drug, in order to further deepen its understanding, we still included this article in the meta-analysis. The age of patients ranged from 1 to 30 years. In the included OLE studies, some participants were given a concomitant administration of two to five antiseizure medications with the intervention, mainly valproate, topiramate, clobazam, stiripentol, and levocetirizine.

**TABLE 1:** Characteristics of included OLE studies.

Study, year	Location	ASM	No. of patients (Male/Female)	Age: mean (SD)	Median treatment duration	Observation period	Dosages (per day)	Concomitant ASMs	Withdrawn
Scheffer et al. (2021)	Israel,Australia, the United Kingdom, Spain, Netherlands, America, France, Poland	Cannabidiol	156/159	9.7 (4.4)	444 days	156 weeks	22 mg/kg	VPA,CLB,STP, LEV,TPM	143
Iannone et al. (2021)	Italy	Cannabidiol	49/44	21.4 (13.5)	8.7 months	12 months	25 mg/kg	VPA, CLB,LEV,STP	29
Devinsky et al. (2019)	America, Europe, Israel	Cannabidiol	133/131	9.8 (4.4)	274 days	48 weeks	21 mg/kg	VPA,CLB,STP, LEV,TPM	75
Sullivan et al. (2020)	North America Europe, Australia	Fenfluramine	128/104	9.1 (4.7)	256 days	24 months	0.2 mg/kg, 0.4 mg/kg, 0.7 mg/kg	VPA, CLB, TPM, LEV, STP	22
Specchio et al. (2020)	Italy	Fenfluramine	29/24	8.6 (4.1–13.9)	9 months	3 to 6 months	0.2∼0.7 mg/kg	VPA,CLB,STP, LEV,TPM	0
Bishop et al. (2021)	Asia, Europe,America	Fenfluramine	34/24	11 ± 4 (5–18)	≥ 1 year	≥ 1 year	0.2 mg/kg	VPA, CLB, TPM, LEV	0
Inoue and Ohtsuka (2015)	Japan	Stiripentol	15/9	5.7 ± 4.3 22.8 ± 1	16 weeks	20 weeks	50 mg/kg	CLB,VPA	0
Myers et al. (2018)	Australia, the United Kingdom	Stiripentol	23/18	5.7 (4.7–9.7)	37 months	12 years	67 mg/kg	VPA,CLB, TPM, LEV	12
Inoue et al. (2009)	Japan	Stiripentol	7/18	6 months	≥8 weeks	≥12 weeks	50∼100 mg/kg	VPA	6
Scheffer et al. (2021)	America	Soticlestat	16	unknow	55 days	4 months	≤600 mg/	VPA,CLB,STP, LEV,TPM	2

Abbreviations: ASM, antiseizure medication; STP, stiripentol; CLB, clobazam; LEV, levetiracetam; SD, standard deviation; TPM, topiramate; VPA, valproate.

**FIGURE 2:**
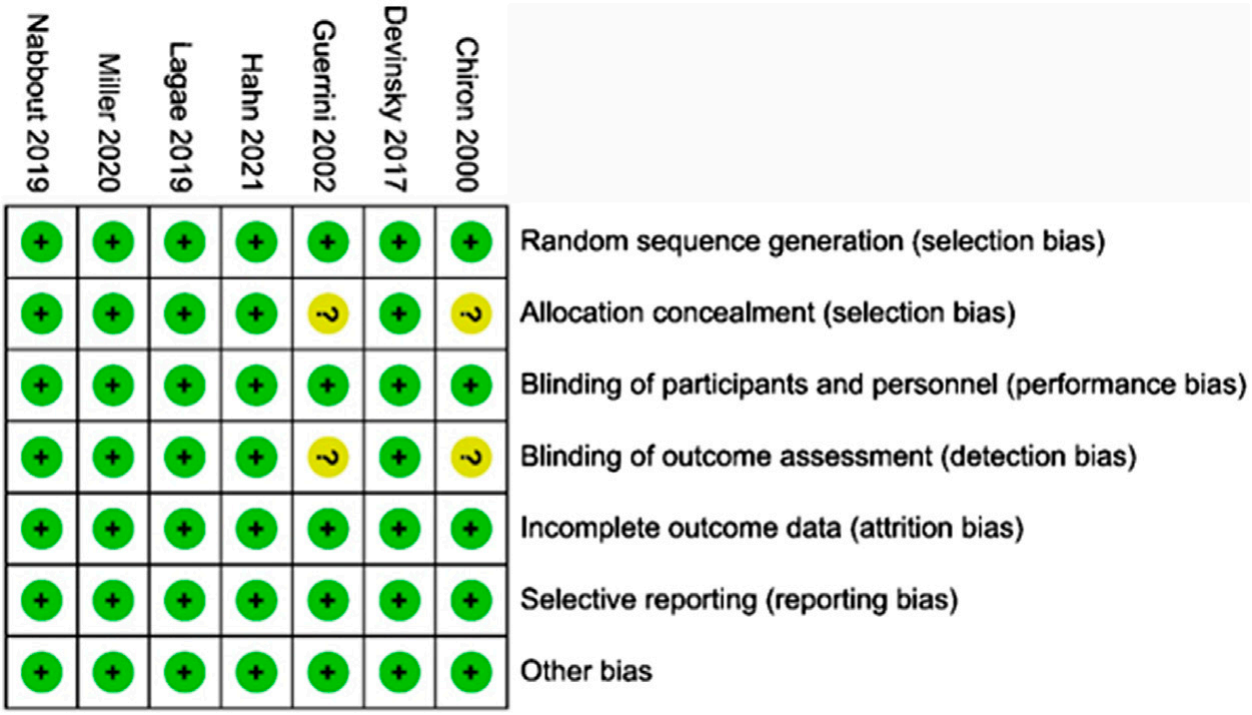
Risk of bias graph.

The main characteristics of the included RCTs are presented in Table 2. Among these, two studies on stiripentol were included (Chiron et al., 2000; Hahn et al., 2022), and a total of 33 patients were randomly selected to receive the stiripentol treatment; two RCTs on cannabidiol were included (Devinsky et al., 2017; Miller et al., 2020), with a total of 194 patients receiving the cannabidiol treatment; two studies of fenfluramine were included (Lagae et al., 2019; Nabbout et al., 2020), with a total of 122 patients receiving fenfluramine; and one study of soticlestat was included (Hahn et al., 2021), with 24 patients receiving soticlestat. Each drug was compared with a placebo in all experiments, and 261 patients were randomly assigned to the placebo group. The age of patients ranged from two to 18 years, with an average age of approximately 9 years. The baseline period of the included RCTs varied from four to 6 weeks. In the included RCTs, some participants were given a concomitant administration of two to five antiseizure medications with the intervention, mainly valproate, topiramate, clobazam, stiripentol, and levocetirizine. Details of concomitant dietary therapy and vagus nerve stimulation are also mentioned in a study by Devinsky et al. (2017). A qualitative assessment was performed by assessing various indicators for each study using the Cochrane tool for the risk of bias (Devinsky et al., 2017; Lagae et al., 2019; Miller et al., 2020; Nabbout et al., 2020; Hahn et al., 2021). Overall, of the seven RCTs included in this study, five were considered to have a low risk of bias. The other two studies on stiripentol (Chiron et al. (2000) and Hahn et al. (2022)) were described as randomized, double-blind trials and blinded to participants and study performers; these lacked sufficient information on allocation concealment and blinded data handlers and were thus considered as uncertain with regard to risk of bias. The results of the risk of bias assessment in the literature are shown in Figure 2.

**TABLE 2:** Characteristics of included RCTs.

Study	Year	Location	ASM	No. of patients intervention/Placebo		Age: mean (SD)	Sex: Male/Female	Baseline period	Double-blind period	Dosages (per day)	Concomitant ASMs	Risk of bias
Chiron et al. (2000), STICLO-France	2000	France	Stiripentol	21	20	9.4 (11.6)	17/24	1 month	2 months	50 mg/kg	VPA, CLB	unknown
Hahn et al. (2022), STICLO-Italy	2002	Italy	Stiripentol	11	11	9.1 (4.0)	11/11	1 month	2 months	51 mg/kg	VPA, CLB	unknown
Devinsky et al. (2017), GWPCARE1	2017	Europe, United States America	Cannabidiol	61	59	9.8 (4.8)	62/58	4 weeks	14(2 + 12)	20 mg/kg	VPA, CLB, TPM, LEV, STP	Low
Miller et al. (2020), GWPCARE2	2020	Asia, Europe, United States America	Cannabidiol	67/66	65	9.4 (4.4)	88/44	4 weeks	14(2 + 12)	20 mg/kg 10 mg/kg	VPA, CLB, TPM, LEV, STP	Low
Lagae et al. (2019)	2019	Asia, Europe, United States America	Fenfluramine	40/39	40	9 (4.7)	54/36	6 weeks	14(2 + 13)	0.7 mg/kg 0.2 mg/kg	VPA, CLB, TPM, LEV	Low
Nabbout et al. (2020)	2019	Europe, United States America	Fenfluramine	43	44	9.1 (4.8)	50/37	6 weeks	15(3 + 12)	0.4 mg/kg	VPA, CLB, TPM, LEV, STP	Low
Hahn et al. (2021), ELEKTRA	2021	Asia, Europe, North America, Australia, Israel	Soticlestat	24	22	9.5 (4.0)	unknown	1 month	20(8 + 12)	≤600 mg	unknown	Low

Abbreviations: ASM, antiseizure medication; STP, stiripentol; CLB, clobazam; LEV, levetiracetam; SD, standard deviation; TPM, topiramate; VPA, valproate.

### Effectiveness comparison

**FIGURE 3:**
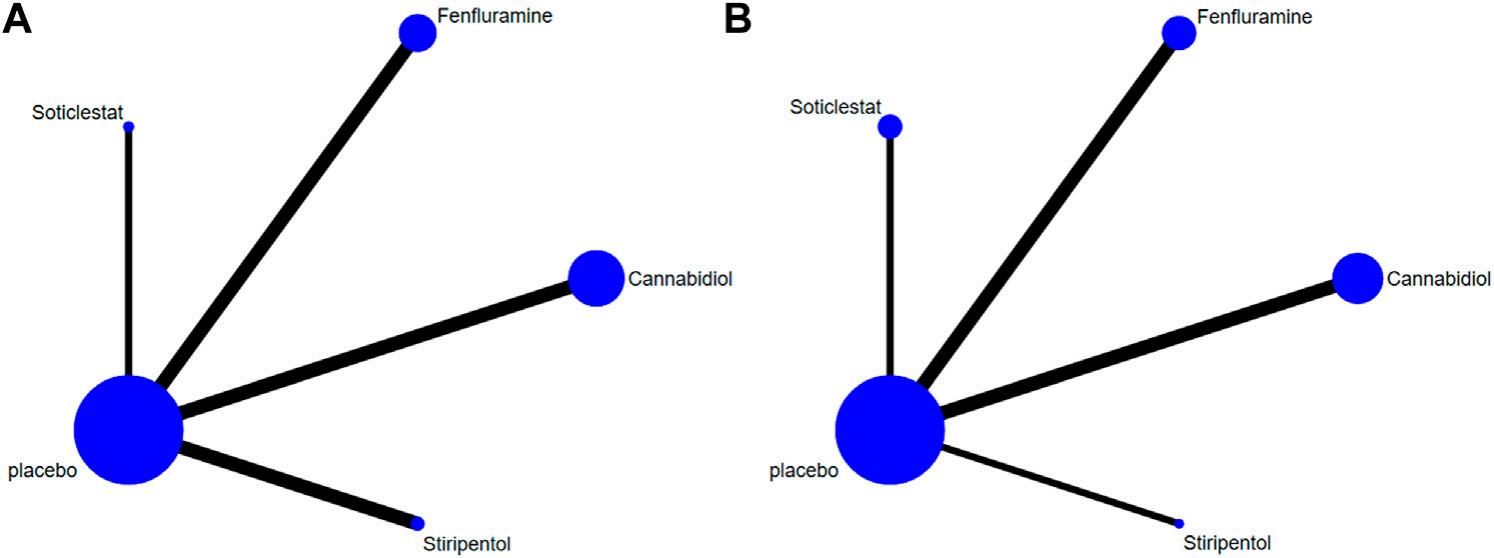
Network plots of treatment comparisons for the efficacy outcomes. Circle size is proportional to the number of study participants assigned to receive each intervention. The line width corresponds to the number of studies comparing the treatments. **(A)** At least 50% reduction of drop seizures; **(B)** Adverse profiles.

**FIGURE 4:**
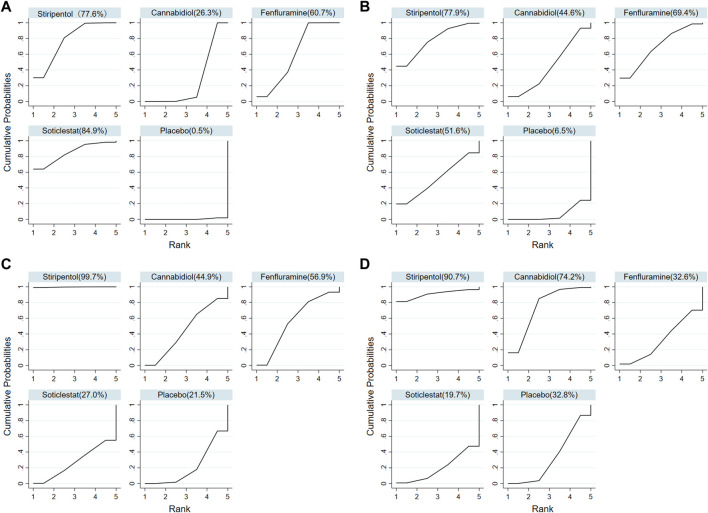
Surface under the cumulative ranking curve probabilities for the ranking. **(A)** At least 50% reduction of drop seizures among treatments; **(B)** Nearly 100% reduction; **(C)** Adverse events; **(D)** Serious adverse events.

**FIGURE 5:**
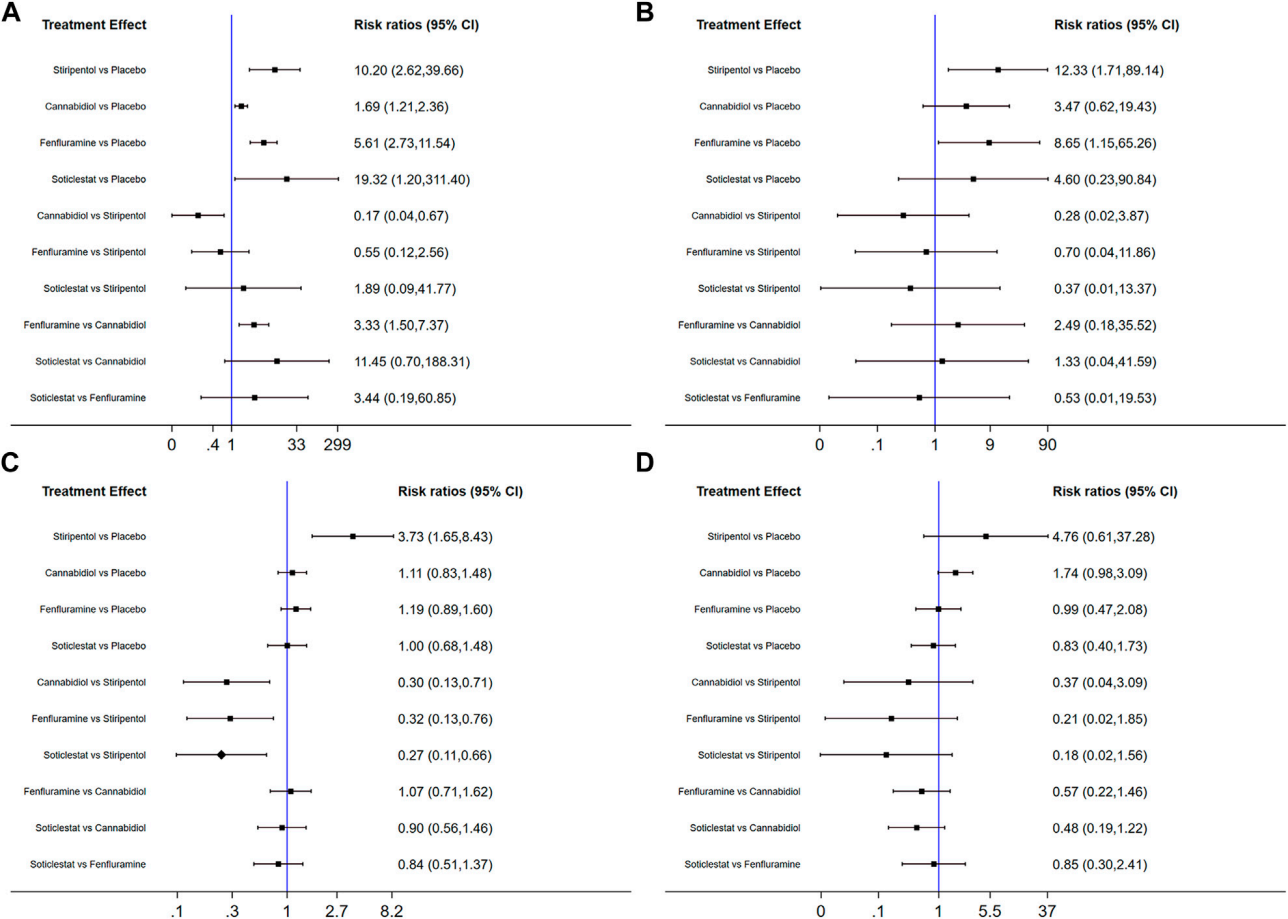
Forest plot of response comparison among treatments. **(A)** At least 50% reduction of drop seizures among treatments; **(B)** Nearly 100% reduction; **(C)** Adverse events; **(D)** Serious adverse events. CI, confidence interval.

**FIGURE 6:**
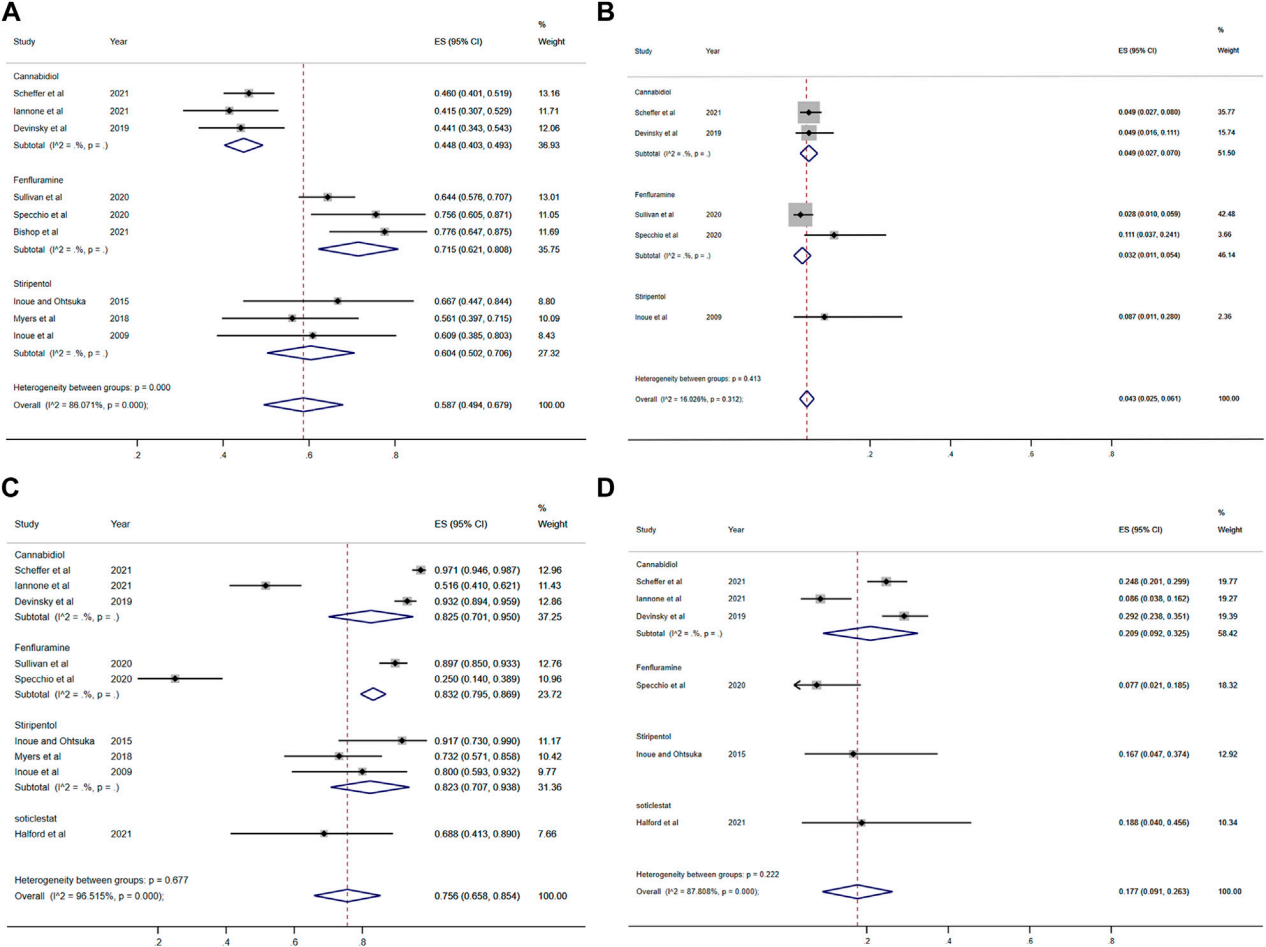
Forest plot of long-term effect and safety comparison among treatments. **(A)** At least 50% reduction of seizures among treatments; **(B)** Nearly 100% reduction; **(C)** Adverse events; **(D)** Serious adverse events. CI, confidence interval.

Among the seven included RCTs, four antiseizure medications were tested. The network plots of patients with a reduction in the convulsive-seizure frequency of at least 50% and the occurrence of AEs are shown in Figure 3. Because all RCTs were compared with a placebo and there were no direct comparisons between antiseizure medications, we could not examine the inconsistencies between direct and indirect treatments. The SUCRA indicated that all antiseizure medications were significantly more effective than the placebo when compared with a baseline seizure reduction ≥50%; soticlestat (84.9%) ranked the highest, followed by stiripentol (77.6%), fenfluramine (60.7%), and cannabidiol (26.3%). Among the included RCTs, a total of 186/373 (49.87%) participants in the intervention groups [soticlestat: *n* = 10/24 (41.67%); stiripentol: *n* = 23/33 (69.70%); fenfluramine: *n* = 65/122 (53.28%); and cannabidiol: *n* = 88/194 (45.36%)] and 42/261 (16.09%) participants in the placebo group achieved at least a 50% reduction in the convulsive seizure frequency from baseline. At the same time, the efficacies of stiripentol (RR: 0.17; 95% CI:0.04, 0.67) and fenfluramine (RR: 3.33; 95% CI: 1.50, 7.37) were significantly higher than that of cannabidiol. Stiripentol (77.9%), fenfluramine (69.4%), and soticlestat (51.6%) had the highest ranking probability compared with baseline seizure reductions of nearly 100%, as shown in Figure 4. All four antiseizure medications were significantly more effective than the placebo. The seven RCTs reported this outcome, with 34/373 (9.12%) participants from the intervention groups [soticlestat: *n* = 2/24 (8.33%); stiripentol: *n* = 13/33 (39.39%); cannabidiol: *n* = 8/194 (4.12%); and fenfluramine: *n* = 11/122 (9.02%)], and 1/261 (0.38%) participants from the placebo group achieving nearly 100% seizure reduction. There were significant differences in the efficacy of stiripentol (RR: 12.33; 95% CI: 1.71, 89.14) and fenfluramine (RR: 8.65; 95% CI: 1.15, 62.26) compared with that of the placebo in achieving complete seizure control. The corresponding forest plots are shown in Figure 5. A meta-analysis of the ten included OLE studies was conducted, and the long-term outcomes with a ≥50% reduction in seizure frequency from baseline as outcome indicators were ranked as fenfluramine(0.715; 95% CI: 0.621, 0.808), stiripentol (0.604; 95% CI: 0.502, 0.706), and cannabidiol (0.448; 95% CI: 0.403, 0.493). Long-term outcomes with a nearly 100% reduction in seizure frequency from baseline as outcome measures were ranked as stiripentol (0.087; 95% CI: 0.011, 0.280), cannabidiol (0.049; 95% CI: 0.027, 0.070), and fenfluramine(0.032; 95% CI: 0.011, 0.054). Since the sample size of stiripentol is too small, the accuracy of the results will be affected. Since the single-group rate is only a descriptive result, not a difference comparison result, there are no so-called “positive” results or results that are statistically significant. The corresponding forest plots are shown in Figure 6.

### Safety comparison

All seven RCTs included in the network meta-analysis provided details on AEs and SAEs, which showed that the incidence of adverse reactions caused by all antiseizure medications was significantly higher than that caused by placebo. The order of the possibility of AEs caused by each antiseizure medication was stiripentol (99.7%), fenfluramine (56.9%), cannabidiol (44.9%), and soticlestat (27.0%). Among the included RCTs, 379/408 (92.89%) patients experienced treatment-emergent AEs in the intervention group [soticlestat: *n* = 66/71 (92.96%); stiripentol: *n* = 21/21 (100%); fenfluramine: *n* = 117/122 (95.90%); and cannabidiol: *n* = 175/194 (90.21%)] compared with 240/298 (80.54%) in the placebo group. Meanwhile, the incidence of AEs caused by stiripentol was significantly higher than that caused by fenfluramine (RR: 0.32; 95% CI: 0.13, 0.76), cannabidiol (RR: 0.30; 95% CI: 0.13, 0.71), and soticlestat (RR: 0.27; 95% CI: 0.11, 0.66). When comparing the incidence of SAEs, only soticlestat (19.7%) was significantly lower than the placebo (32.8%). The order of the possibility of SAEs caused by the other antiseizure medications was stiripentol (90.7%), cannabidiol (74.2%), and fenfluramine (32.6%). During the treatment period, 71/408 (17.40%) participants in the intervention groups [soticlestat: *n* = 11/71 (15.49%); stiripentol: *n* = 5/21 (23.81%); fenfluramine: *n* = 15/122 (12.30%); and cannabidiol: *n* = 40/194 (20.62%)] and 38/298 (12.75%) participants in the placebo group experienced serious treatment-emergent adverse events. However, there were no significant differences among the antiseizure medications. The corresponding forest plots are shown in Figure 5. A meta-analysis of ten OLE studies was conducted. A ranking based on long-term outcomes of incidence of AEs was assigned, ordered as fenfluramine(0.832; 95% CI: 0.795, 0.869), cannabidiol (0.825; 95% CI: 0.701, 0.950), stiripentol (0.823; 95% CI: 0.707, 0.938) and soticlestat (0.688; 95% CI: 0.413, 0.890). A ranking based on long-term outcomes of the incidence of SAEs was assigned, ordered as cannabidiol (0.209; 95% CI: 0.092, 0.325), soticlestat (0.188; 95% CI: 0.040, 0.456), stiripentol (0.167; 95% CI: 0.047,0.374), and fenfluramine(0.077; 95% CI: 0.021, 0.185). Since the sample size of stiripentol and soticlestat are too small, the accuracy of the results will be affected. The corresponding forest plots are shown in Figure 6.

## Discussion

Dravet syndrome is a rare, severe form of hereditary epilepsy. It has attracted much attention in epileptic encephalopathy and is one of the most clinically challenging epilepsy syndromes (Selvarajah et al., 2021). The persistent threat of intractable seizures, multiple comorbidities, and premature death severely affects the quality of life of children and their families (Mudigoudar et al., 2016; Lagae et al., 2018). Epilepsy mortality in DS is 5.1 times higher than that in adults with refractory epilepsy (Harris et al., 2015). The main causes of increased mortality in patients with DS are status epilepticus, seizure-related complications, and sudden epileptic death (Sharawat et al., 2021). Currently, many treatment options are available for DS. The three primary forms of treatment available are antiseizure medications, dietary modifications (usually a ketogenic diet), and surgical intervention (Chin et al., 2021). Nevertheless, antiseizure medications remain the mainstream treatment for epilepsy (Chin et al., 2021). The British National Institute of Clinical Practice guidelines and Chinese epilepsy diagnosis and treatment guidelines recommend valproic acid, topiramate, and/or clobazam as first-line treatment drugs for DS; stiripentol, levetiracetam, and zonisamide can be used as additional therapeutic drugs, while sodium channel blockers are not recommended for the treatment of DS (Nunes et al., 2012). Because DS has no specific symptoms in the early stage, it is often misdiagnosed as other types of epilepsy, and the application of sodium channel inhibitors (carbamazepine, oxcarbazepine, and lamotrigine) and benzodiazepines may promote encephalopathy and worsen the condition (Strzelczyk and Schubert-Bast, 2022). Despite reasonable multidrug therapy and ketogenic diets, approximately 45% of children experience more than four seizures per month due to limited drug options for DS (Wheless et al., 2020). Therefore, there is an urgent need to identify new drugs for DS treatment. In recent years, with the development of large-scale clinical trials, significant breakthroughs have been made in the development of DS treatment drugs. The U.S. FDA approved stiripentol and cannabidiol in 2018, and fenfluramine in 2020 for the treatment of DS (Lattanzi et al., 2018; Yıldız et al., 2019; Sharawat et al., 2021). In 2021, it was found that soticlestat, a new drug for the treatment of DS, showed good safety and effectiveness in the treatment of DS.

A series of recently published randomized controlled trials reveals growing interest in the role of stiripentol, fenfluramine, cannabidiol, and soticlestat in DS treatment. Two double-blind, randomized, placebo-controlled trials of stiripentol as an adjuvant therapy for valproic acid and clobazam in France [STICLO-France (Chiron et al., 2000)] and Italy [STICLO-Italy (Hahn et al., 2022)] showed that DS patients over 3 years of age showed better responses to these drugs than placebo in both the ≥50% reduction in seizure frequency and complete control of seizures. The initial dose of stiripentol was 15–20 mg/(kg d), which was increased to a target dose of 50 mg/(kg d) in two to 4 weeks. The maximum dose for children can be as high as 100 mg/(kg d). The main adverse reactions included drowsiness, fatigue, ataxia, appetite loss, and liver damage (Jullien et al., 2015).

The Nordic Cochrane Centre, T.C.C, 2020, Devinsky et al., 2017 [GWPCARE1 (Devinsky et al., 2017)] and Miller et al., 2020 [GWPCARE2 (Miller et al., 2020)] had investigated the efficacy and safety of cannabidiol for the treatment of DS. The results showed that the effective rate of cannabidiol in patients with refractory DS was significantly higher than that in the placebo group (42.6–49.3% vs. 26.2–27.1%), while in the subgroup that used clobazam at the same time, the effective rate was higher (47.5–62.5% vs. 23.7–36.6%). The initial dose of cannabidiol was 2–5 mg/(kg·d), and the maximum dose was 25 mg/(kg·d), according to attack and tolerance. Adverse reactions included drowsiness, fatigue, diarrhea, and anorexia (Devinsky et al., 2015).

In recent years, two double-blind, randomized controlled trials [Lagae 2019 (Lagae et al., 2019) (without the concurrent use of stiripentol) and Nabbout 2020 (Nabbout et al., 2020) (with the concurrent use of stiripentol)] have confirmed the effectiveness and safety of fenfluramine in the treatment of DS. The percentage of patients with seizures that decreased by ≥50% was significantly higher than that of the placebo group (68–38% vs. 5–12%). The recommended dose of fenfluramine is 0.12–0.90 mg/(kg·d) (Ceulemans et al., 2012). Low-dose fenfluramine is generally well-tolerated in the treatment of DS. The most common adverse reactions were loss of appetite and weight loss. Other common adverse reactions include diarrhea, fatigue, lethargy, fever, and upper respiratory tract infections (Strzelczyk and Schubert-Bast, 2022).

Soticlestat (≤600 mg/day weight-adjusted) is a new drug used for the treatment of DS. It showed better efficacy and safety in a recently completed phase 2 clinical trial, Elektra. Recruitment to phase 3 clinical trials is ongoing. The main adverse reactions were lethargy and constipation (Hahn et al., 2021).

With the advent of these RCTs, different mechanisms of action, different administered doses, and AEs have led to difficulties in the selection of clinically appropriate drugs. Therefore, it is important to determine the efficacy and safety of these four antiseizure medications (stiripentol, fenfluramine, cannabidiol, and soticlestat) in DS patients. Thus, the current study included the seven studies described above and a network meta-analysis of the efficacy and safety of these drugs in the hope of providing a reference for clinicians when choosing treatments.

The drug comparison demonstrated that all four antiseizure medications resulted in a more significant reduction in convulsive-seizure frequency than the placebo among patients with DS. Soticlestat was the most efficacious adjunctive therapy, followed by fenfluramine, and stiripentol. However, since the soticlestat sample size was very small, this result needs further verification. We hope that soticlestat will achieve good results in future clinical trials. Stiripentol was the only medication with a statistically significant difference in the incidence of AEs compared to the placebo. In addition, because stiripentol has the highest probability of AEs and SAEs, fenfluramine is a better choice for DS treatment. This result is consistent with the conclusions of Devi et al. (2021). There were no significant differences in the effects of the four antiseizure medications in achieving complete control of epilepsy.

However, clinical decisions depend on all available information, and the RCTs included in our study were only short-term trials, which would affect the accuracy of the conclusions. To further determine the efficacy and safety of these four antiseizure medications, we included post-commercial studies or open-label RCTs that provided important information on long-term efficacy and safety for a meta-analysis. In this study, soticlestat was included one article, while each of stiripentol, fenfluramine and cannabidiol were included three articles. Due to the small sample size of soticlestat and the lack of corresponding efficacy outcome indicators, the long-term efficacy and safety of soticlestat need further study. In addition to soticlestat, the comprehensive safety and efficacy can be concluded that fenfluramine is the first choice, followed by stiripentol. This is consistent with our conclusion.

In addition, it is also important to consider drug-drug interactions between antiseizure medications. Because it will affect the efficacy and safety of antiseizure medications. When stiripentol is combined with fenfluramine, the maximum plasma concentration and systemic exposure of fenfluramine are significantly increased, so the dose of fenfluramine can be appropriately reduced (Boyd et al., 2019). Valproic acid does not impact on stiripentol pharmacokinetics (PK), and some data suggest that clobazam may increase stiripentol concentrations by around 25% (Peigné et al., 2018). Studies have shown that fenfluramine had no significant effect on the PK of valproic acid, stiripentol, clobazam, or nor-clobazam (the major and active metabolite of clobazam), and thus no dose adjustments are needed for these commonly prescribed anticonvulsant medications when administered with fenfluramine (Balagura et al., 2020). When clobazam is coadministered with cannabidiol, the area under the curve (AUC_0-t_) of the active cannabidiol metabolite 7-hydroxy-cannabidiol increases 50% (Franco and Perucca, 2019). It has already been demonstrated that concomitant clobazam affects cannabidiol’s safety profile and raises the likelihood of adverse events, particularly somnolence, drowsiness, and pneumonia (Lattanzi et al., 2020). Therefore, dose reductions of clobazam may be considered based on a benefit-risk assessment (Morrison et al., 2019)**.** Based on PK alone, dose adjustments are not likely to be necessary when cannabidiol is given concomitantly with stiripentol or valproic acid (Morrison et al., 2019). Coadministration of stiripentol with cannabidiol resulted in a slight increase in stiripentol exposure, but this is unlikely to be clinically relevant, so dose adjustment of stiripentol may not be necessary. Cannabidiol had no significant effect on the PK of valproic acid (Morrison et al., 2019). So far, the clinical relevance of these interactions remains largely unknown, and so will have some impact on our findings.

To date, epilepsy control in DS remains a great challenge, and no single drug treatment is highly effective for this syndrome (Wirrell, 2016). Our study showed that soticlestat is likely to be a better adjunctive antiseizure medication in terms of combined efficacy and safety. However, the accuracy of the results needs to be verified further because of the insufficient number of patients evaluated. In addition, owing to the lack of RCTs for a direct comparison, the present analysis only provides indirect comparisons from methodological RCTs, which may differ from reality. Therefore, while our findings provide a reference for clinicians, in practical clinical applications, clinicians need to consider each patient and their situation individually and weigh the advantages and disadvantages of each treatment before administering drugs. In other words, the ranking of treatments in this study is based on the mean SUCRA value but does not imply that a higher-ranked treatment is substantially better than a lower-ranked treatment. Differences in the number of trials per comparison and effect sizes between treatments probably affected ranking probabilities. Furthermore, considering the small number of patients in a single experiment for some drugs and the large confidence intervals for the efficacy and safety outcomes, the results of the final network meta-analysis should be interpreted with caution.

Our study has some limitations that need consideration. First, the sample size was small and some drugs were tested on fewer than 100 participants. Second, because DS is a relatively rare disease with a limited number of related RCTs, some of the included RCTs were performed more than a decade ago. Third, the mean age of the study population was approximately 9 years, which may have further confounded the results due to the effects of age and regression to the mean. Fourth, most of the included studies used only seizures as the main result. However, DS includes multiple types of convulsive seizures, and non-convulsive seizures are associated with DS. The number of included studies was small, and although a systematic literature search was conducted, the bias of the small-study effects persisted. Finally, the shorter treatment periods (up to 2 months) in each study may not be sufficient to assess the true longer-term efficacy of each drug, and more direct clinical trials (head-to-head comparisons) are needed to further validate these results.

## Conclusion

The efficacy of all four antiseizure medications was significantly higher than that of the placebo. The SUCRA ranking showed that stiripentol was the most likely to cause AEs and SAEs. Cannabidiol was slightly inferior to the other three antiseizure medications in terms of efficacy and safety. The results of this indirect comparison suggest that soticlestat, fenfluramine, and stiripentol may have little difference in efficacy, but soticlestat and fenfluramine are safer. Regarding comprehensive efficacy and safety, soticlestat may be the best adjuvant therapy for DS, followed by fenfluramine. This conclusion is consistent with the comparison of long-term efficacy and safety. However, soticlestat has only recently passed phase 2 clinical trials, and the results are yet to be verified. We look forward to achieving positive results in subsequent clinical trials. Nonetheless, this still provides a reference for the clinical selection of the four antiseizure medications. We hope there will be more clinical, drug interaction, and pharmacoeconomic studies related to DS in the future, which will provide more scientific references for the selection of therapeutic drugs for patients with DS in clinical practice.

## Data Availability

The original contributions presented in the study are included in the article/supplementary material, further inquiries can be directed to the corresponding authors.
